# College campus smoking policies and programs and students' smoking behaviors

**DOI:** 10.1186/1471-2458-5-74

**Published:** 2005-07-07

**Authors:** Tyrone F Borders, K Tom Xu, Donna Bacchi, Lee Cohen, Danielle SoRelle-Miner

**Affiliations:** 1 Department of Health Policy and Management, University of Arkansas for Medical Sciences, Little Rock, Arkansas, USA; 2 Division of Health Services Research, Texas Tech University Health Science CenterLubbock, Texas, USA; 3 Department of Pediatrics and Center for Tobacco Control and Prevention, Texas Tech University Health Science Center, Lubbock, Texas, USA; 4 Department of Psychology, Texas Tech University, Texas, USA; 5 Center for Tobacco Control and Prevention, Texas Tech University Health Science Center, Lubbock, Texas, USA

## Abstract

**Background:**

Although tobacco use in the United States has declined over the past 20 years, cigarette use among college students remains high. Additional research is thus needed to determine how university tobacco control policies and preventive education programs affect college students' smoking behaviors.

**Methods:**

Approximately 13,000 undergraduate students at 12 universities or colleges in the state of Texas completed a web-based survey. College smoking policies were obtained from a survey of college administrators and from college websites. Logistic regression analyses were conducted to estimate the effects of individual smoking policies and programs on the odds of cigarette smoking.

**Results:**

Of the individual programs, only having a preventive education program on campus was associated with lower odds of smoking. The existence of smoking cessation programs and designated smoking areas were associated with higher odds of smoking. Policies governing the sale and distribution of cigarettes were insignificantly associated with smoking.

**Conclusion:**

Rather than focusing on policies restricting cigarette sales and use, college administrators should consider implementing or expanding tobacco prevention and education programs to further reduce student smoking rates.

## Background

Despite reductions in smoking rates over the past 20 years, tobacco use remains a key public health concern. According to recent estimates from the Centers for Disease Control, smoking is the leading cause of preventable death in the United States [1]. Cigarette use among college students is of particular concern as they have a substantially higher prevalence of smoking relative to the general adult population [1,2].

To curb smoking rates on college campuses, several national organizations recommend that colleges and universities enact stricter tobacco control and prevention policies. The American College Health Association (ACHA) and American Cancer Society (ACS) advocate that colleges ban smoking in all campus buildings and residence halls; prohibit the sale, sampling, and advertising of tobacco products; restrict smoking to a minimum of 20 feet from building entrances and air intake units; limit or prohibit spit tobacco use on campus; and implement tobacco prevention/education and cessation programs on campus [3,4].

Many institutions have responded to these recommendations. According to a survey of 50 colleges and universities across the United States, the number of campuses regulating cigarette use in student housing increased from 1% to 54% between 1994–1995 and 2002–2003 [5]. A cross-sectional survey of college students suggests that campus housing smoking bans are effective in decreasing smoking rates [6]. In addition to policies regulating where students can smoke, many universities have implemented public education campaigns aimed at non-smokers and smoking cessation programs targeting current smokers. According to a survey of health directors at 393 four-year universities across the U.S., 40% of schools offered some type of smoking cessation program [6]. Several studies have shown that anti-smoking messages can reduce smoking rates among younger adults [7-10], but little research has been conducted to assess their effectiveness among college students.

While there are some indications that more stringent tobacco policies and greater availability of promotion and prevention programs reduce smoking rates on college campuses, there is a lack of conclusive research in the area. More extensive regulatory policies could have a negative effect if they elicit a rebellious response from students. An additional question which remains insufficiently answered is the degree to which regulatory policies as compared to prevention or education are associated with smoking behaviors. An improved understanding of the relative effects of different types of policies and programs could assist college and university administrators as they consider how to invest resources to curb smoking.

## Methods

### Survey sample

A web-based survey, which was approved by Texas Tech University Health Sciences Center's institutional review board (IRB), was conducted among college students in Texas. The survey contained questions covering past and current cigarette, cigar, and smokeless tobacco use; knowledge of the risks associated with tobacco use; and responses to tobacco marketing campaigns. The sampling frame and methodology have been discussed elsewhere [11], but are briefly described here. All 72 colleges and universities within the state of Texas were invited to participate in a web-based survey of their undergraduate students' tobacco use. Of the 72 institutions, 27 did not elect to respond to requests to participate in the survey, 3 did not collect e-mail addresses from their students (which was necessary to recruit students), and 29 refused to release students' e-mail addresses. Thus, 13 college and universities agreed to participate in the study, representing 184,559 students.

Students from the participating schools were e-mailed and invited to participate in a web-based survey about tobacco use. To encourage participation, students were offered to enroll in a lottery to receive one of five $500 airline gift certificates upon completion of the survey. They were then directed to a web site where they could complete the survey. Approximately 13% of students from the 13 participating schools completed the survey. For the analyses conducted here, one college was excluded because it had a particularly low response rate (approximately 2%). Thus, the final sample represented 13,041 undergraduate students at 12 four-year colleges and universities in Texas.

### Variables

Because we were concerned with the occurrence of smoking over a defined time period, as opposed to the quantity of cigarette consumption, we defined current smoking as having smoked at least one cigarette in the past 30 days. This is the standard method of categorizing persons as current vs. non-current smokers [6,12-14].

College-level campus smoking policies and regulations were obtained through mail surveys of college administrators. College administrators were asked whether their school had specific campus policies which restricted tobacco distribution, prohibited tobacco sales, restricted smoking to 20 feet from building entrances, prohibited smoking in student residence halls or dormitories, clearly identified non-smoking areas, prohibited tobacco ads in campus publications, prohibited events sponsored by tobacco companies, and prohibited smoking in all indoor public areas. They were also asked whether their school provided preventive education related to smoking and smoking cessation courses. Data collected from college administrators were supplemented by information obtained from each college's website.

Student-level control variables included the smoking status of a student's social circle (roommates and friends), academic information (college and academic classification), demographics (age, race/ethnicity, and gender), and memberships in certain groups (fraternity/sorority and intercollegiate sports team).

### Analyses

To investigate whether universities' smoking policies and programs were significantly associated with the probability of smoking, multivariate logistic regressions were conducted. Among the ten policies, three (prohibition of tobacco advertisements in campus publications, events sponsored by tobacco companies, and smoking in indoor public areas) were present at all universities in the sample. Due to the lack of variation in these three policies, they were excluded from the analyses.

Univariate logistic regression analyses were first conducted to estimate unadjusted odds ratios for the university-level policies and programs. Student-level variables were included in the final multivariate model to control for their potential confounding effects. Because more than one student from each participating university was present in the sample, a traditional regression analysis was not used as it would provide larger standard errors of the estimated coefficients. To correct for the within-cluster dependence of the observations, a sandwich-like robust estimator of the variance-covariance matrix was used.

## Results

The sample is described in Table 1. Seven of the 12 schools (58.3%) had a policy regarding tobacco distribution and prohibited smoking in residence halls. Only 2 (16.7%) universities prohibited tobacco sales on campus and had restrictions on smoking to a minimum of 20 feet from entrances of buildings. Five schools (41.7%) clearly identified non-smoking areas. Five schools (41.7%) provided preventive education and four (33.3%) delivered smoking cessation courses.

**Table 1:** Descriptive Statistics of Sample

*College-level policies and programs*	% (n = 12)
Restrict tobacco distribution	58.3
Prohibit tobacco sales on campus	16.7
Restrict smoking to 20 ft. from entrances	16.7
Prohibit smoking in residence halls	58.3
Clearly identify non-smoking areas	41.7
Provide prevention and education	41.7
Provide smoking cessation classes	33.3
	
*Student-level control variables*	% (n = 13,041)
Age group (referent = 18–19)	
20–21	38.5
≥22	38.3
Race/Ethnicity (referent = white)	
Hispanic	11.7
Black	2.6
Others	11.4
Gender (referent = male)	
Female	61.3
Roommate smokes (referent = no roommate)	
Yes	27.8
No	50.4
% of friends who smoke (referent = ≤20%)	
21–50%	28.0
≥50%	15.5
College (referent = applied science)	
Arts	15.7
Business	18.2
Engineering	8.6
Human Sciences	11.8
Mass Communications	11.7
Others	21.7
Academic classification (referent = freshman)	
Sophomore	14.7
Junior	20.1
Senior	28.1
Graduate	22.2
Fraternity/sorority member (referent = no)	
Yes	16.2
Intercollegiate athlete (referent = no)	
Yes	4.6

Approximately 37% of the students in the sample reported smoking in the past 30 days. The majority of the sample (74%) was non-Hispanic white and about 61% was female. Although about half of the sample had roommates who smoked, only 16% reported that more than half of their friends were smokers. Approximately 16% of the students were members of fraternities or sororities. Fewer than 5% were members of intercollegiate sports teams.

Table 2 shows the effects of universities' smoking policies and programs on the probability of smoking, controlling for students' characteristics. Only three of the seven policies or programs showed significant effects. Preventive education programs decreased the odds of smoking by about 23%. In contrast, smoking cessation programs and the identification/designation of smoking areas significantly increased the odds of smoking by 30% and 45%, respectively.

**Table 2:** Unadjusted and Adjusted Odds Ratios (OR) for Current Smoking

	Unadjusted OR (95% CI)	*p*	Adjusted OR (95% CI)	*p*
*College-level policies and programs*				
Restrict tobacco distribution	1.12 (0.82–1.52)	0.48	1.00 (0.90–1.11)	0.97
Prohibit tobacco sales on campus	0.82 (0.67–1.02)	0.07	0.95 (0.83–1.08)	0.43
Restrict smoking to 20 ft. from entrances	0.83 (0.67–1.03)	0.09	0.91 (0.79–1.04)	0.15
Prohibit smoking in residence halls	1.21 (0.94–1.54)	0.14	1.02 (0.91–1.36)	0.77
Clearly identify non-smoking areas	1.48 (1.21–1.81)	<0.01	1.45 (1.32–1.60)	<0.01
Provide prevention and education	0.95 (0.66–1.39)	0.81	0.77 (0.63–0.93)	<0.01
Provide smoking cessation classes	0.95 (0.66–1.37)	0.80	1.30 (1.17–1.45)	<0.01
				
*Student-level control variables*				
Age group (referent = 18–19)				
20–21	-		0.92 (0.84–1.01)	0.07
≥22	-		0.85 (0.74–0.98)	0.02
Race/Ethnicity (referent = white)				
Hispanic	-		0.85 (0.72–1.00)	0.05
Black	-		0.51 (0.37–0.69)	<0.02
Others	-		0.94 (0.87–1.01)	0.11
Gender (referent = male)				
Female	-		0.94 (0.90–0.99)	0.02
Roommate smokes (referent = no roommate)				
Yes	-		1.42 (1.26–1.59)	<0.01
No	-		0.67 (0.64–0.70)	<0.01
% of friends who smoke (referent = ≤20%)				
21–50%	-		2.62 (2.46–2.79)	<0.01
≥50%	-		6.44 (5.45–7.60)	<0.01
College (referent = applied science)				
Arts	-		1.02 (0.83–1.27)	0.84
Business	-		0.91 (0.79–1.03)	0.14
Engineering	-		0.80 (0.73–0.89)	<0.01
Human Sciences	-		0.96 (0.82–1.12)	0.62
Mass Communications	-		0.99 (0.85–1.15)	0.88
Others	-		1.18 (1.02–1.37)	0.03
Academic classification (referent = freshman)				
Sophomore	-		0.86 (0.74–0.99)	0.04
Junior	-		0.79 (0.67–0.93)	0.01
Senior	-		0.79 (0.72–0.86)	<0.01
Graduate	-		0.59 (0.50–0.69)	<0.01
Fraternity/sorority member (referent = no)				
Yes	-		1.09 (0.98–1.22)	0.12
Intercollegiate athlete (referent = no)				
Yes	-		0.93 (0.80–1.07)	0.30

Estimation results indicated that student characteristics played a more important role in predicting smoking status. The smoking status of a students' social circle was significantly associated with smoking status. Also, maturity decreased the probability of being a smoker. As compared with freshmen, sophomores, juniors, seniors, and graduate students were significantly less likely to smoke. This pattern is mirrored in the age variable.

As compared with non-Hispanic white students, Hispanic and black students were less likely to smoke. Also, females were less likely to smoke, which is consistent with national level observations among adults [1]. Being a member of a fraternity/sorority or intercollegiate sports teams was not found to significantly contribute to the probability of smoking.

## Discussion

Restriction of tobacco distribution, prohibition of tobacco sales, restriction of smoking within 20 feet from entrances, and prohibition of smoking in residence halls were not associated with the odds of smoking, suggesting that they are ineffective in influencing college students' smoking behaviors. Yet, other programs do appear to impact college students' smoking behaviors. We found that students enrolled at schools with smoking cessation programs had higher smoking rates, but this should not necessarily lead to the conclusion that smoking cessation classes contribute to smoking. It is reasonable to infer that administrators of colleges and universities that have higher smoking rates implement cessation programs in an attempt to reduce cigarette use. On the other hand, it has been shown that that college campus smoking cessation courses and programs are underutilized and that college students often want to quit on their own [15].

Prevention-oriented education was associated with lower odds of current cigarette use. Students who were exposed to education about smoking were 23 percent less likely to smoke compared their counterparts who were not exposed to campus education. There is little information about the efficacy of such campaigns on college campuses, but a number of studies have shown that anti-tobacco messages have a positive effect on tobacco use rates in other populations [7,9,10]. When Massachusetts evaluated the effects of its comprehensive state-wide tobacco control program on college students who were in high school when the program was initiated, results indicated that those students exposed to the tobacco prevention and education portions of the program had lower smoking rates than college students that were not exposed [14].

Although several of the policies and programs examined in this paper do not appear to affect current cigarette use, it is possible that others do affect college students' smoking status. Taking into consideration that all of the schools in the present sample prohibited tobacco advertisements in campus publications, events sponsored by tobacco companies, and smoking in indoor public areas, it was not possible to test for their effects on the odds of smoking. The latter three regulations may limit smoking to a point where the addition of other regulations has little impact. It should also be noted that the policies and programs discussed in the present paper could yield other, unmeasured benefits, such as reducing smoking rates among faculty and staff.

Despite the notable findings, the present study has a number of methodological limitations. Although the data set includes a large number of respondents, the participation rate could bias the results. The level of distribution and enforcement of policies was not measured, but could influence their effectiveness in controlling tobacco use [16]. Additional research is thus warranted to determine the degree to which college students are aware of published campus tobacco policies. Because we did not obtain detailed descriptions of the nature and content of preventive education and smoking cessation courses available on campus, we can not conclude whether particular programs are more effective than others.

## Conclusion

In summary, results from the present study imply a need for additional evaluation of the effects of various approaches to tobacco control and prevention on student tobacco usage rates. Future studies should employ different methodologies whereby distinct conclusions about the effectiveness of specific policies or programs can be determined. In the interim, however, the present results suggest that prevention and education programs may have the strongest protective effect on college students' cigarette use.

## Competing interests

The author(s) declare that they have no competing interests.

## Authors' contributions

TFB conceived the study design, participated in data analysis, and led the writing of the manuscript. KTX assisted with data analysis and contributed to the writing of the manuscript. DB was responsible for conceiving the survey and overseeing data collection. LC assisted with the survey design and data collection. DSM assisted with data collection. All authors contributed to the writing of the manuscript.

## Pre-publication history

The pre-publication history for this paper can be accessed here:


